# Features of severe asthma in young children from Romania

**DOI:** 10.1186/2045-7022-3-S1-P22

**Published:** 2013-05-03

**Authors:** Mihai Craiu, Iustina Violeta Stan

**Affiliations:** 1 Institute for Mother and Child Care, Carol Davila Medical University, MedLife Children’s Hospital, Romania; 2 Institute for Mother and Child Care, Carol Davila Medical University, Respiratory Diseases, Romania

## Background and aim

Asthma is one of the most important chronic disease in children, due to high prevalence and increased direct and indirect costs. A complex effort [GA2LEN, Me-DALL and ARIA groups] was organized to address the area of severe allergic diseases, including severe asthma. The concept of “problematic-severe-asthma” has risen and two major groups of asthmatic children were identified: difficult-to-treat asthma and therapy resistant asthma. There were published few papers on asthmatic children from Romania, only one paper addressing the area of severe asthma in school-age children. We describe features of severe asthma in young and preschool children from Romania.

## Materials and methods

Cross-sectional study of patients in tertiary-referral asthma clinic: were included children with previously diagnosed asthma or recurrent-wheezing phenotype, up-to 10-years of age. All these children were referred by GP or pediatrician because of severe/uncontrolled disease. A complex evaluation was performed to exclude alternative diagnostics. At first visit, an extensive training for device use, inhalation technique and trouble-shooting was implemented.

## Results

313 referrals (between Oct 2011-Mar 2012) were evaluated. 233 children were evaluated 1-5 times. 216 children were included. They were 56.7 months old (4-125 mo), 153 (70.83%) were boys, 182 (84.26%) were inner-city children. For 202 (93.53%) evaluation per-protocol was completed. 153 (75.74%) had identifiable factors for not achieving control. 49 (24.26%) had severe disease that generated multiple visits or exacerbations. In 6 children (12.24%) asthma was excluded. The remaining 43 (87.76%) had uncontrolled asthma, but really difficult-to-treat or refractory asthma was documented only in 8 (3.7% of included patients, 16.33% of children with more severe disease and no identifiable factors). They didn’t present male dominance (50% girls) were significantly older (83.6 months) had frequently severe rhino-conjunctivitis (75%) and atopic dermatitis (62.5%). In 2 cases severe side effects of medication were documented (severe depression associated with LTRA). High frequency of exacerbations (87.5% with 1-4 episodes) was noted.

## Conclusions

1. Problematic-severe-asthma is more frequent than previously described in romanian children (3.7% vs 1.5%). 2. They are older, more often girls, have severe allergic associated-diseases and exacerbate more frequent than other asthmatic children in spite of aggressive treatment.

